# Evolutionary Trajectory of the Tet(X) Family: Critical Residue Changes towards High-Level Tigecycline Resistance

**DOI:** 10.1128/mSystems.00050-21

**Published:** 2021-05-18

**Authors:** Chao-Yue Cui, Qian He, Qiu-Lin Jia, Cang Li, Chong Chen, Xiao-Ting Wu, Xiao-Jing Zhang, Zhuo-Yu Lin, Zi-Jian Zheng, Xiao-Ping Liao, Barry N. Kreiswirth, Ya-Hong Liu, Liang Chen, Jian Sun

**Affiliations:** a Center for Emerging and Zoonotic Diseases, College of Veterinary Medicine, South China Agricultural University, Guangzhou, China; b Guangdong Laboratory for Lingnan Modern Agriculture, South China Agricultural University, Guangzhou, China; c Guangdong Key Laboratory for Veterinary Drug Development and Safety Evaluation, College of Veterinary Medicine, South China Agricultural University, Guangzhou, China; d National Risk Assessment Laboratory for Antimicrobial Resistance of Animal Original Bacteria, College of Veterinary Medicine, South China Agricultural University, Guangzhou, China; e Center for Discovery and Innovation, Hackensack Meridian Health, Nutley, New Jersey, USA; f Department of Medical Sciences, Hackensack Meridian School of Medicine, Nutley, New Jersey, USA; g Institutes of Agricultural Science and Technology Development, Yangzhou University, Yangzhou, China; California State University, Fresno

**Keywords:** tigecycline resistance, Tet(X), key residue changes, evolutionary path, *Riemerella anatipestifer*

## Abstract

The emergence of the plasmid-mediated high-level tigecycline resistance mechanism Tet(X) threatens the role of tigecycline as the “last-resort” antibiotic in the treatment of infections caused by carbapenem-resistant Gram-negative bacteria. Compared with that of the prototypical Tet(X), the enzymatic activities of Tet(X3) and Tet(X4) were significantly enhanced, correlating with high-level tigecycline resistance, but the underlying mechanisms remain unclear. In this study, we probed the key amino acid changes leading to the enhancement of Tet(X) function and clarified the structural characteristics and evolutionary path of Tet(X) based upon the key residue changes. Through domain exchange and site-directed mutagenesis experiments, we successfully identified five candidate residues mutations (L282S, A339T, D340N, V350I, and K351E), involved in Tet(X2) activity enhancement. Importantly, these 5 residue changes were 100% conserved among all reported high-activity Tet(X) orthologs, Tet(X3) to Tet(X7), suggesting the important role of these residue changes in the molecular evolution of Tet(X). Structural analysis suggested that the mutant residues did not directly participate in the substrate and flavin adenine dinucleotide (FAD) recognition or binding, but indirectly altered the conformational dynamics of the enzyme through the interaction with adjacent residues. Matrix-assisted laser desorption ionization–time of flight mass spectrometry (MALDI-TOF MS) and UV full-wavelength scanning experiments confirmed that each mutation led to an increase in activity without changing the biochemical properties of the Tet(X) enzyme. Further phylogenetic analysis suggested that Riemerella anatipestifer served as an important incubator and a main bridge vector for the resistance enhancement and spread of Tet(X). This study expands the knowledge of the structure and function of Tet(X) and provides insights into the evolutionary relationship between Tet(X) orthologs.

**IMPORTANCE** The newly emerged tigecycline-inactivating enzymes Tet(X3) and Tet(X4), which are associated with high-level tigecycline resistance, demonstrated significantly higher activities in comparison to that of the prototypical Tet(X) enzyme, threatening the clinical efficacy of tigecycline as a last-resort antibiotic to treat multidrug-resistant (MDR) Gram-negative bacterial infections. However, the molecular mechanisms leading to high-level tigecycline resistance remain elusive. Here, we identified 5 key residue changes that lead to enhanced Tet(X) activity through domain swapping and site-directed mutagenesis. Instead of direct involvement with substrate binding or catalysis, these residue changes indirectly alter the conformational dynamics and allosterically affect enzyme activities. These findings further broaden the understanding of the structural characteristics and functional evolution of Tet(X) and provide a basis for the subsequent screening of specific inhibitors and the development of novel tetracycline antibiotics.

## INTRODUCTION

Antibiotic resistance is recognized as a major global health issue and poses a high health care burden in both developed and developing countries (1). Tetracyclines are one of the most successful antibiotics in the past 80 years, due to their excellent antibacterial activity and oral benefits, and have been widely used in the treatment of human and animal infections or as feed additives for animal growth (2). Tigecycline is among the most effective broad-spectrum tetracyclines, and it is regarded as the last-line-of-defense drug for the treatment of multidrug-resistant (MDR) Gram-negative bacterial infections. However, the recently reported mobile tigecycline resistance mechanism Tet(X) threatens the therapeutic utility of tigecycline (and that of other newer-generation tetracyclines, e.g., eravacycline, omadacycline, and sarecycline) from an already shrinking antibiotic arsenal.

Tet(X) is a flavin-dependent monooxygenase capable of catalyzing regioselective hydroxylation of tetracyclines to 11a-hydroxy-tetracyclines in the presence of molecular oxygen and NADPH (3). Tet(X) was originally found in obligate anaerobic bacteria (Bacteroides), although the hydroxylation reaction of Tet(X) requires the participation of molecular oxygen. Two other Tet(X) orthologs, Tet(X1) and Tet(X2), were also identified in Bacteroides spp., located on the conjugative transposon of Tn*4351*, although Tet(X1) is a truncated variant and did not have enzymatic activity (3, 4). In 2007, Ghosh and coworkers described a *tet*(X)-containing Sphingomonas strain from a soil sample as the first identification of *tet*(X) gene in aerobic bacteria (5). Subsequently, in 2013, *tet*(X) and its orthologs were detected in a variety of clinical pathogenic bacteria, including Klebsiella pneumoniae, Escherichia coli, Pseudomonas aeruginosa, etc., from a hospital in Sierra Leone (6). However, these variants [e.g., Tet(X) and Tet(X2)] only conferred resistance to the early tetracyclines, such as tetracycline, oxytetracycline, doxycycline, etc. with limited activity against tigecycline (7).

In 2019, two novel plasmid-encoded Tet(X) orthologs, namely Tet(X3) and Tet(X4), which mediated high levels of tigecycline resistance, were discovered in E. coli and Acinetobacter isolates from human and food animal samples, representing a growing threat to the latest-generation tetracyclines (8, 9). To date, five new Tet(X) orthologs, designated Tet(X3), Tet(X4), Tet(X5), Tet(X6), and Tet(X7), have been reported in a variety of clinical pathogenic and environmental bacteria (10, 11). Worrisomely, Tet(X) orthologs conferring high-level tigecycline resistance have also been found in clinical carbapenem- or colistin-resistant strains harboring *bla*_NDM-1_ or *mcr-1* (8, 9, 12). Compared with the previously reported Tet(X), Tet(X3) to Tet(X7) showed 86%, 96%, 90%, 84%, and 84% amino acid identities, respectively, but their activities are significantly enhanced (8, 9, 11, 13–15).

Several residues, including H231 and M372 in the tetracycline binding region and E43, R114, and D308 in the flavin adenine dinucleotide (FAD) binding region, have been considered key sites underlying the activity of Tet(X4) (16). However, the residues were 100% conserved across all Tet(X) orthologs, which cannot explain the high-level resistance among those newer variants. In this study, we performed domain swapping and site-directed mutagenesis on Tet(X2) and identified several key residue changes leading to the increase of Tet(X2) activity. Based on structural analysis, we further explored the mechanisms of how these mutations affect Tet(X2) activity. Last, we used phylogenetic analysis reconstruct the molecular evolution of Tet(X) and found that Riemerella anatipestifer served as an important incubator and a main bridge vector for the resistance enhancement and spread of Tet(X).

## RESULTS

### Characterization of the Tet(X) family.

**FIG 1:**
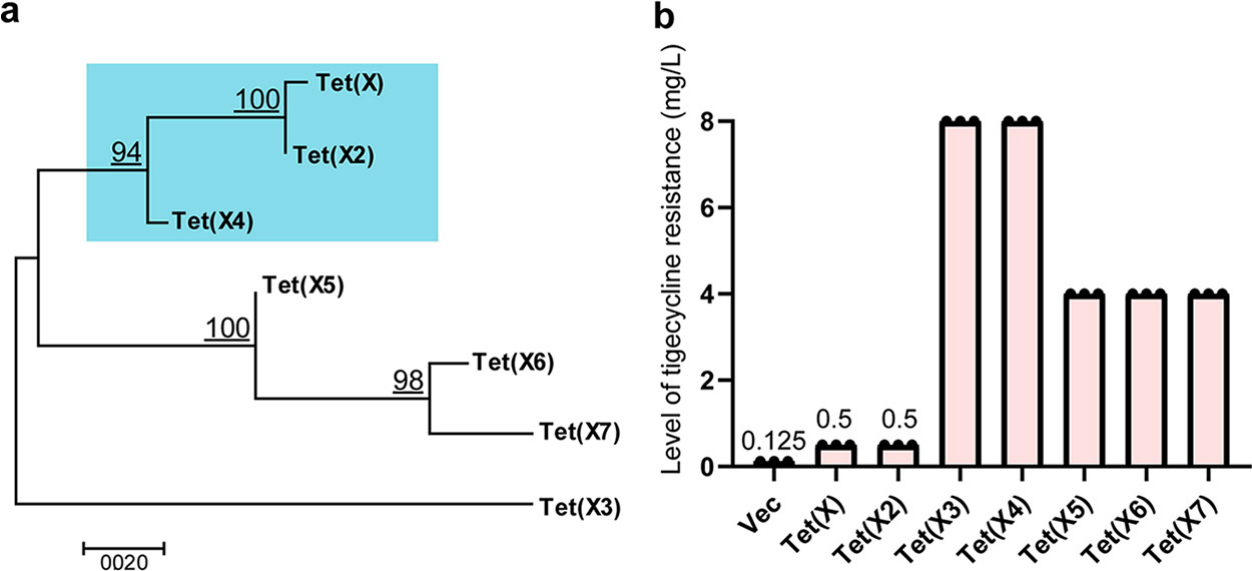
Characteristics of 7 reported orthologs of Tet(X). (a) The phylogenetic relationship of the seven orthologs. The Tet (X4) and Tet(X/X2) orthologs were located in the same clade and are marked with blue shading. (b) An antimicrobial susceptibility test was used to determine the levels of tigecycline resistance mediated by the 7 orthologs. The experiments were repeated three times. Vec, empty vector.

To characterize the relationship between different Tet(X) orthologs, a phylogenetic tree was constructed with MEGA 7 using the maximum-likelihood method. The Tet(X) phylogenetic tree, consisting of 7 reported orthologs, was separated into three monophyletic clades (Fig. 1a). The originally reported Tet(X/X2) and the newly discovered Tet(X4) showed the highest similarity and were colocated in the same clade. Among them, Tet(X/X2) exhibited a wide host range and was present in a variety of bacteria, including Bacteroides spp., Delftia spp., Enterobacteriaceae, Riemerella anatipestifer, Parabacteroides spp., etc., while Tet(X4) was occasionally detected in Aeromonas caviae and Acinetobacter spp. (17, 18) but mainly found in E. coli (9, 19, 20).

We subcloned the full-length sequences of the 7 reported *tet*(X) genes into the E. coli expression system and examined tigecycline susceptibility. The results showed that Tet(X3) and Tet(X4) had the highest level of tigecycline resistance (8 mg/liter), followed by Tet(X5), Tet(X6), and Tet(X7) (4 mg/liter), whereas Tet(X) and Tet(X2) had the lowest tigecycline MICs (0.5 mg/liter) (Fig. 1b). Note that the Tet(X4) had the highest relatedness with Tet(X2), with 96% amino acid identity (only 22 amino acid differences). To this end, we started with the two orthologs to probe the key residue changes leading to enhanced Tet(X) activity.

### Molecular docking analysis.

To explore the structure difference of Tet(X), we performed sequence alignments and homology modeling of Tet(X) orthologs. By comparing the X-ray crystal structure of Tet(X2) with the homology model of Tet(X4), we found that low-activity Tet(X2) and high-activity Tet(X4) shared a highly conserved substrate and FAD binding cavity, and the substrate docking poses were also highly similar (see Fig. S1 in the supplemental material). Indeed, all amino acid residues located within 4 Å of the binding substrate and the FAD coenzyme were identical (see Fig. S2 in the supplemental material). Consequently, our results suggested that the amino acid changes underlying increased Tet(X) activity were not located in the substrate or FAD binding regions.

10.1128/mSystems.00050-21.2 FIG S1 (a) An enlarged view of the docking posture of tigecycline in the tigecycline binding cavity (b) An enlarged view of the docking posture of flavin adenine dinucleotide (FAD) molecules in the FAD binding cavity. The tigecycline and FAD molecules are shown as hot pink and grey sticks, respectively. Modelling of Tet(X2) was derived from the crystal structure (PDB identifier 4A6N), while modelling of Tet(X4) was based on the Tet(X2) structure using Modeller v9.24. Download FIG S1, TIF file, 1.7 MB. Copyright © 2021 Cui et al. 2021 Cui et al. https://creativecommons.org/licenses/by/4.0/ This content is distributed under the terms of the Creative Commons Attribution 4.0 International license.

10.1128/mSystems.00050-21.3 FIG S2 Sequence alignment of 8 Tet(X) variants using ESPript v3.0. Secondary structure elements of Tet(X2) (PDB accession number 4A6N) are depicted above the diagram. Helices are represented by wavy lines, and “TT” denotes turns. The active sites are marked with blue dots below. The residues within 4 Å of tigecycline and FAD molecules are marked with blue and orange dots below, respectively. The 5 key residue changes are marked with purple stars below. Download FIG S2, TIF file, 1.5 MB. Copyright © 2021 Cui et al. 2021 Cui et al. https://creativecommons.org/licenses/by/4.0/ This content is distributed under the terms of the Creative Commons Attribution 4.0 International license.

### Domains swapping and site-directed mutagenesis analysis.

**FIG 2:**
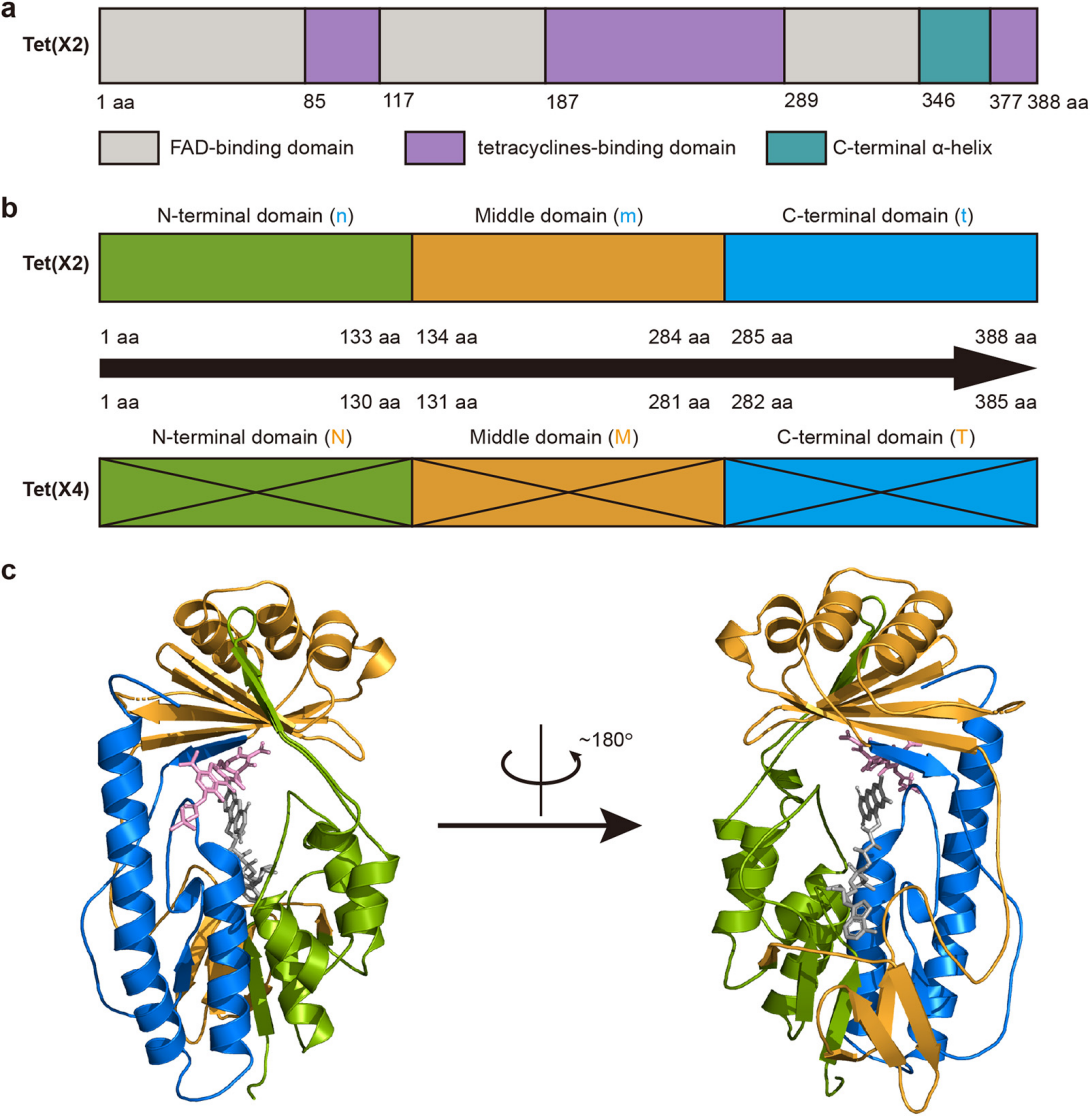
Repartition of Tet(X) based on the protein sequences of Tet(X2) and Tet(X4). (a) The three typical domains of Tet(X2) are colored as depicted by the key. (b) The Tet(X2) and Tet(X4) sequences are divided into three new regions, namely, the N-terminal domain (green), the middle domain (yellow), and the C-terminal domain (blue). The numbers refer to the range of amino acid residues of each of the region. (c) Spatial positions of the three regions in the crystal structure of Tet(X2) (PDB identifier 4A6N). Tigecycline and FAD molecules are shown as pink and gray sticks, respectively.

**FIG 3:**
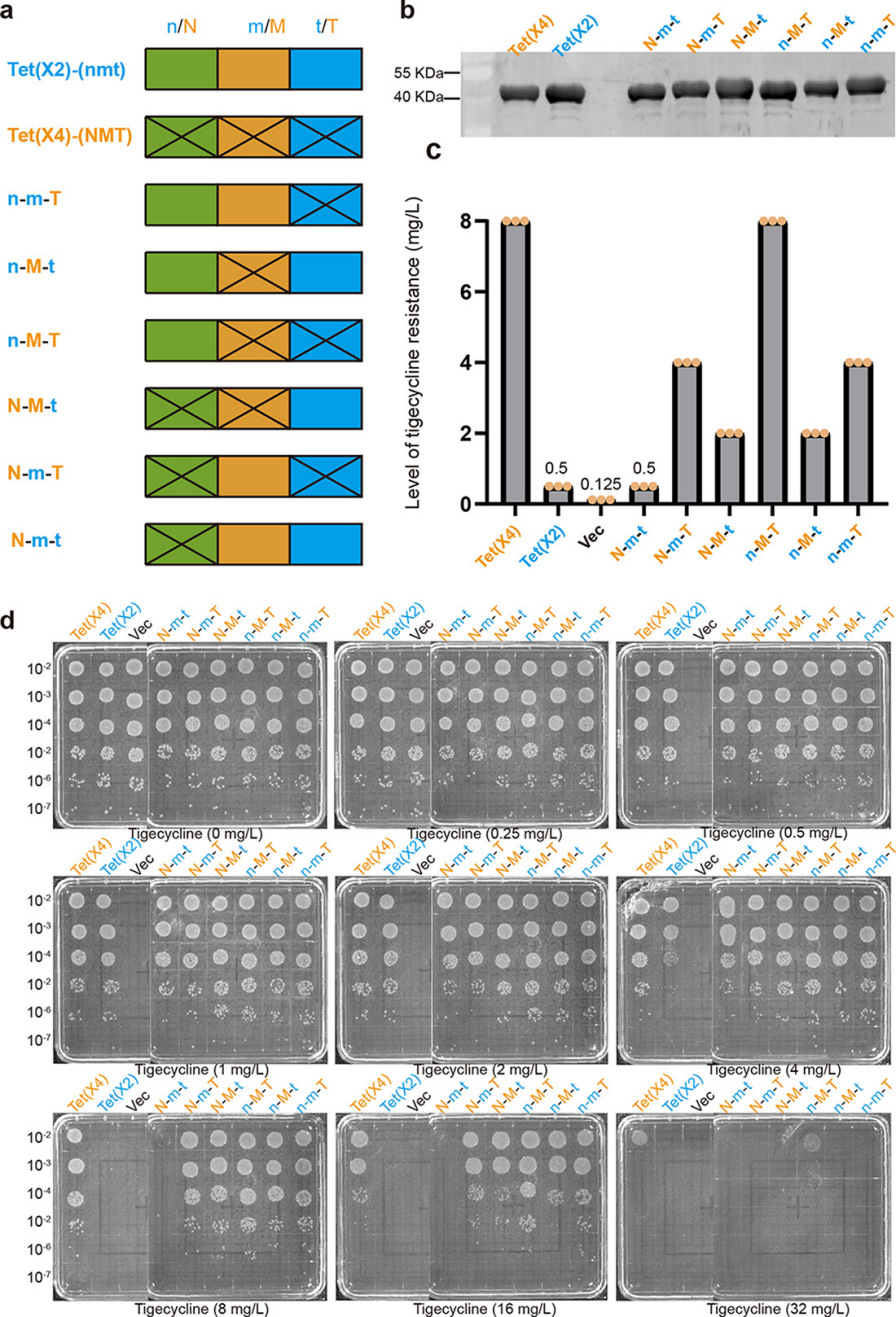
Construction and phenotype verification of recombinant domain proteins. (a) In total, 6 recombinant constructs were obtained by domain swapping. (b) Western blotting was used to determine the expressions of recombinant proteins. (c) MICs determined by the broth microdilution method for Tet(X2), Tet(X4), and its recombinant protein constructs in Escherichia coli JM109. The experiments were repeated three times. (d) Determination of the growth viability of E. coli carrying different recombinant proteins of Tet(X) on the LB agar plates with different levels of tigecycline. n-m-T, a derivative of Tet(X2) with C-terminal region of Tet(X4) in place of its native c-terminal domain; n-M-t, a mosaic version of Tet(X2) whose middle domain was exchanged with that of Tet(X4); n-M-T, a derivative of Tet(X4) with the N-terminal region of Tet(X2) in place of its native N-terminal domain; N-M-t, a derivative of Tet(X4) with the C-terminal region of Tet(X2) in place of its native C-terminal domain; N-m-T, a mosaic version of Tet(X4) whose middle domain was exchanged with that of Tet(X2); N-m-t, a derivative of Tet(X2) with the N-terminal region of Tet(X4) in place of its native N-terminal domain; Vec, empty vector.

We then used domain swapping experiment to explore the domains associated with the increase active of Tet(X4). The structure of Tet(X) protein can be divided into the following three regions: a tetracycline binding region, an FAD binding region, and a C-terminal α-helix connecting the two domains (21). The tetracycline binding domain and FAD binding domain of Tet(X) each comprised 3 noncontiguous regions (Fig. 2a). For convenience, we divided Tet(X2) into three domains based on the amino acid sequences, namely, the N-terminal domain (amino acids [aa] 1 to 133), the middle domain (aa 134 to 284), and the C-terminal domain (aa 285 to 388) (Fig. 2b and c). We then performed saturation domain swaps between Tet(X2) and Tet (X4), and a total of 6 constructs were obtained (Fig. 3a). Western blot assays confirmed that all recombinant proteins were well expressed at comparable levels (Fig. 3b). Tigecycline susceptibility testing revealed that the interchange of the C-terminal or the middle domain alone led to significant elevation in activity of Tet(X2), with the MIC increased by 8- and 4-fold, respectively. If both the C-terminal and the middle domains were swapped simultaneously, the activity of Tet(X2) was restored to the same level as that of Tet(X4), indicating that the two domains have a superimposed effect on the activity of Tet(X2) (Fig. 3c and d).

**FIG 4:**
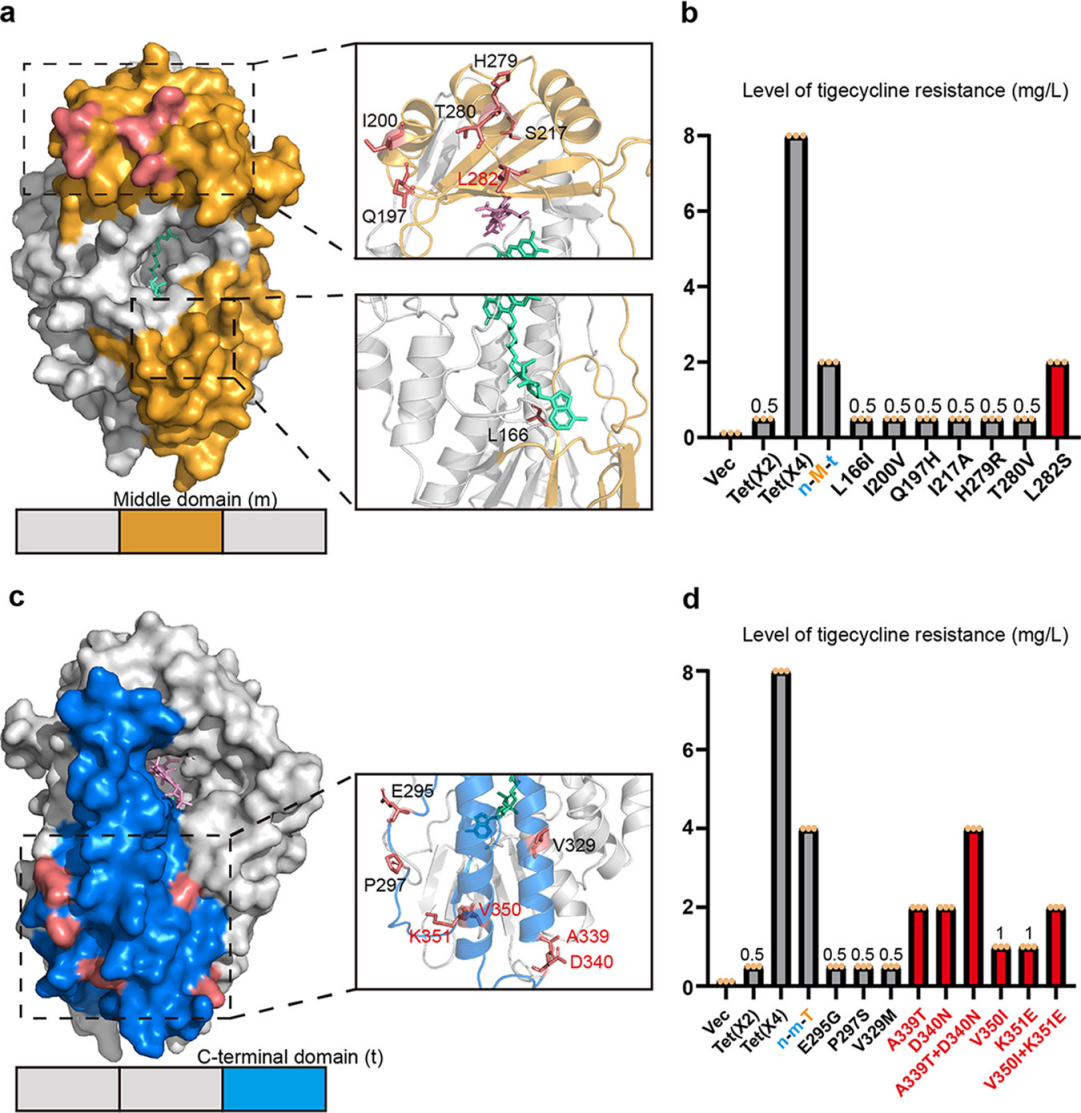
Location and site-directed mutagenesis of residues differing between Tet(X2) and Tet(X4). (a, c) Positions of the differing residues in the middle and C-terminal domains, respectively. (b, d) MICs of tigecycline in different Tet(X) mutant constructs (in E. coli JM109). Red typeface indicates the mutations that lead to increased Tet(X2) activity.

Since the activity of Tet(X2) was mainly affected by the middle and the C-terminal domains, we then used site-directed mutagenesis to probe key residue changes in the two domains. Sequence alignment identified 6 different residues between the middle domains of Tet(X2) and Tet(X4), including L166I, I200V, Q197H, S217A, H279R, T280V, and L282S (Fig. 4a). We successfully constructed all six mutants in E. coli JM109. The susceptibility testing results showed that, compared with the original Tet(X2), the L282S replacement increased the tigecycline MIC by 4-fold (2 mg/liter), reaching the same MIC level as that of the middle domain swapping construct (Fig. 4b). However, the replacement at the remaining 5 sites did not change the MICs, suggesting that residue 282 was the key amino acid in determining the increased Tet(X) activity in the middle domain region.

Similarly, sequence alignment identified 7 residue differences (E295G, P297S, V329M, A339T, D340N, V350I, and K351E) between Tet(X2) and Tet(X4) in the C-terminal domain (Fig. 4c). Susceptibility testing of individual replacement constructs showed that the V350I and K351E replacements conferred a 2-fold increase in tigecycline MICs (1 mg/liter), and the A339T and D340N replacements conferred a 4-fold increase (2 mg/liter), whereas the E295G, P297S, and V329M replacements did not affect MICs. A339T-D340N and V350I-K351E double mutants increased the tigecycline MICs by 8- and 4-fold, respectively (Fig. 4d). Our results indicated the four C-terminal domain residues were involved in the Tet(X) activity changes with additive effects. In total, 5 candidate mutants (L282S, A339T, D340N, V350I, and K351E) were found to be the key residues leading to increased Tet(X) activity. Moreover, multiplex mutants with 5 residue changes can restore the tigecycline MIC to the same level as that of Tet(X4). In addition, we also performed some single and multiple point mutations at the corresponding sites of Tet(X4) and found that the tigecycline MICs of these mutants all decreased to various degrees (Table 1).

**TABLE 1:** MICs of 5 tetracyclines for the study strains.

Strain	MIC (mg/liter)^*a*^				
	TC	MIN	TGC	OMA	ERA
Tet(X2)	8	0.5	0.5	4	0.5
Tet(X4)	64	16	8	32	4
Tet(X2)-L282S	16	2	2	8	1
Tet(X2)-A339T	16	2	2	8	1
Tet(X2)-D340N	16	2	2	8	1
Tet(X2)-V350I	16	2	1	4	1
Tet(X2)-K351E	16	2	1	4	1
Tet(X2)-A339T-D340N	32	4	4	16	1
Tet(X2)-V350I-K351E	32	4	2	8	2
Tet(X2)-L282S-A339T-D340N-V350I-K351E	64	16	8	32	4
Tet(X4)-S279L	32	8	4	16	2
Tet(X4)-T336A-N337D	32	8	4	8	2
Tet(X4)-I347V-E348K	32	8	4	16	2
Tet(X4)-T336A-N337D-I347V-E348K	16	2	2	16	2
E. coli JM109-pBAD24	0.25	0.06	0.125	0.5	<0.03

a TC, tetracycline; MIN, minocycline; TGC, tigecycline; ERA, eravacycline; OMA, omadacycline.

Additional susceptibility testing of the mutants against other tetracycline drugs (tetracycline, minocycline, eravacycline, and omadacycline) showed similar MIC changes (Table 1). The results indicated that these active residue changes, associated with increased tigecycline resistance, did not reduce the ability of the enzyme to modify earlier classes of tetracyclines.

### Functional verification of different Tet(X) mutants.

**FIG 5:**
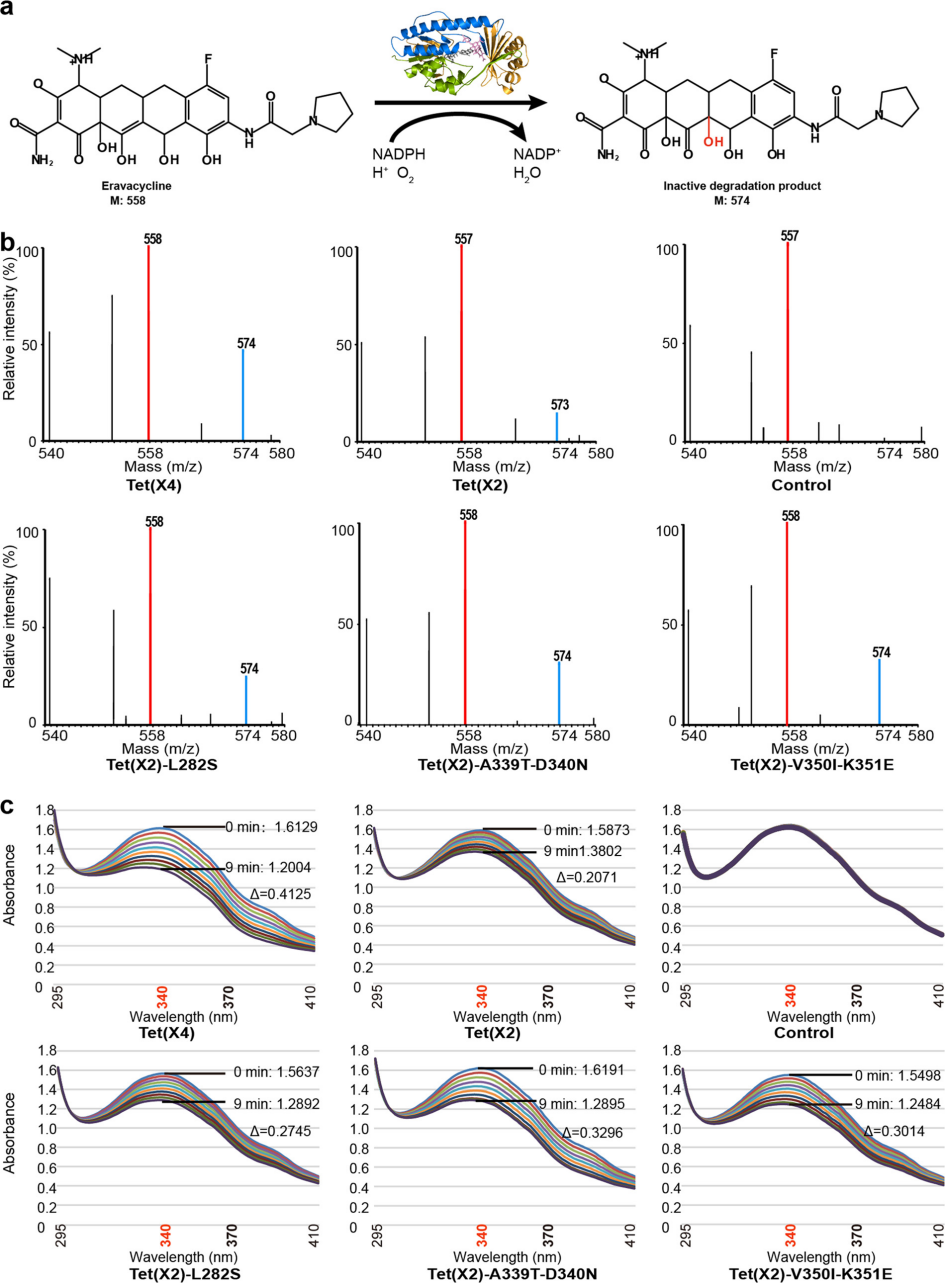
Functional verification of different Tet(X) mutants. (a) The model diagram of Tet(X) degradation of eravacycline. (b) Mass spectrometric determination of monooxygenated products based on matrix-assisted laser desorption ionization–time of flight mass spectrometry (MALDI-TOF MS). The peaks of eravacycline and its monooxygenated products were at 558 ± 1 *m/z* (red line) and 574 ± 1 *m/z* (blue line), respectively. (c) UV-visible spectra of eravacycline catalyzed by different Tet(X) mutants. The maximum absorption peaks of NADPH and eravacycline were at 340 nm and 370 nm, respectively. The Δ symbol indicates the value of the absorbance decrease at 340 nm after 10 min of enzymatic reaction.

We then examined their ability to degrade eravacycline (tigecycline was not used because of its poor stability in the reaction solution). Tet(X2), Tet(X4), and 3 site-directed mutagenesis mutants (L282S, A339T-D340N, and V350I-K351E) were introduced into a BL21(DE3)-pET28a E. coli expression system, and the fusion proteins carrying the 6×His tag were further purified *in vitro*. The purified enzymes were sequentially added to the eravacycline buffer containing Mg^2+^ and NADPH, and the monooxygenated product peak of the drug was detected by mass spectrometry. The results showed that all mutants were accompanied by additional peaks at *m/z* 574 after 15 min of reaction, corresponding with the addition of 16-Da enzymatic inactivation product, as evidenced by monooxygenation (Fig. 5a and b). In addition, the intensities of the product peaks correlated with the levels of tigecycline resistance conferred by different Tet(X) enzymes and mutants, with that of Tet(X4) being the highest, followed by those of the L282S, A339T-D340N, and V350I-K351E mutants, while that of Tet(X2) remained the lowest.

In addition, UV full-wavelength scanning was used to detect the consumption of NADPH and the inactivation of eravacycline, using the same reaction as described above. Eravacycline and NADPH showed absorption peaks at 370 nm and 340 nm, respectively. As shown in Fig. 5c, after the addition of different mutant proteins, the consumption of NADPH by enzymatic reaction resulted in a time-dependent decrease in the intensity of the absorption peak at 340 nm, while the NADPH peak in the control group (without enzyme) did not show apparent changes. Importantly, the NADPH consumption rates among all the mutants were faster than that of the original Tet(X2), which further confirmed that these residue changes lead to the increase of enzyme activities (Fig. 5c).

### Structure-based analysis of the key residue changes.

**FIG 6:**
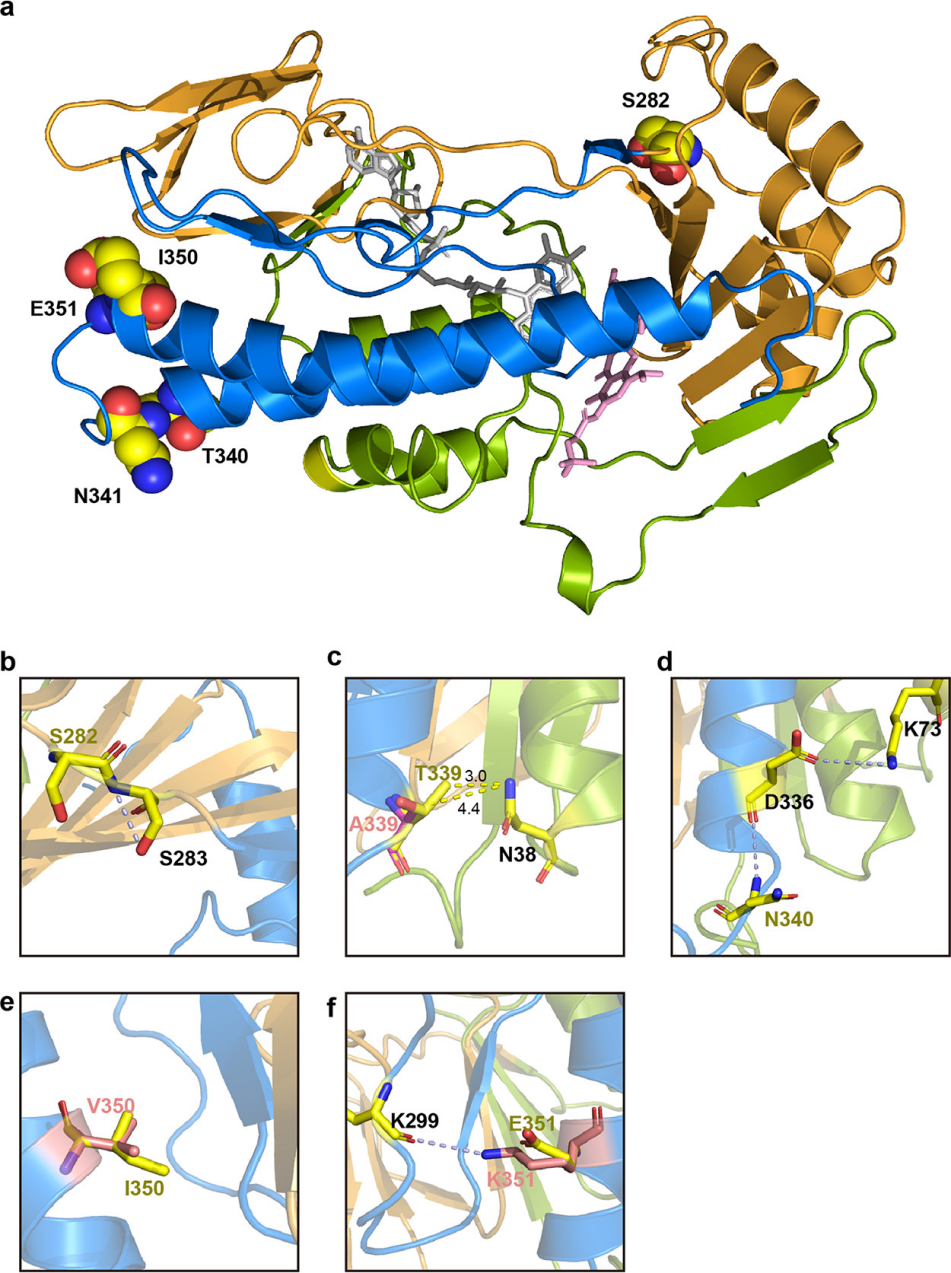
Structural comparison of different Tet(X) mutant residues. (a) Cartoon schematic showing the locations of 5 mutations with increased activity. Residues are shown as spheres, and the carbon, nitrogen, and oxygen atoms are colored in yellow, blue, and red, respectively. (b to f) Interaction between mutant residues and adjacent amino acids. Gray dotted lines represent hydrogen bonds. The number next to the yellow dotted line indicates the shortest distance between two residues.

The combination of domain exchange and site-directed mutagenesis experiments identified five residues (L282S, A339T, D340N, V350I, and K351E) involved in Tet(X2) functional enhancement (Fig. 6a). Among them, the L282 residue was located on the outer surface of the protein, between the α8-helix and the β16-sheet of the tetracycline binding region, and it was close to the FAD hydrophobic pocket. Structure-based analysis showed that when residue 282 was mutated from threonine to serine, it could form a hydrogen bond with the adjacent residue S283, affecting the spatial configuration of β16-sheet and consequently enhancing the interaction between the terminal β17-sheet and FAD (Fig. 6b). Another possible mechanism of the increased activity of residue 282 was that the change of this residue improves the binding efficiency with oxygen and FAD, since it was close to the previously estimated oxygen binding pocket (22). Interestingly, the two adjacent residue changes, T280A and T281A, had also been found to have an enhanced effect on enzyme activities in previous experimental evolution studies (7), suggesting that the vicinity of residue 282 was a critical region for the increased activity and evolution of Tet(X). However, when residue 280 changed from threonine to valine, the enzyme activity did not change, and another mutation in this region, H279R, had the same result. Therefore, although residues 279 and 280 were also located in or near the functional region, the specific amino acid changes at these two positions were not the cause of the difference in activity between Tet(X2) and Tet(X4).

Residues 339 and 340 were located at or close to the α10-helix of Tet(X2). When the 339th residue is changed from alanine to threonine, the steric hindrance between the larger threonine and the residue N38 on the α1-helix increases, and the distance between the two residues is shortened from 3.2 Å to 2.4 Å, forcing the α1-helix to move inward, thereby making FAD form a closer integration with Tet(X) (Fig. 6c). Additionally, residue D336 and residue K73 exhibit hydrogen bond interactions and lock their respective α-helical structures. When residue 340 was mutated from aspartic acid to asparagine, the hydrogen bond (formed by N340 and D336) breaks the locked conformation between D336 and K73, and the α10-helix where D336 was located opens outwards, causing the structure of the FAD binding cavity to change (Fig. 6d).

The residues 350 and 351 were located on the C-terminal α11-helix, connecting the substrate and the FAD binding region. After the 350th residue was mutated from valine to isoleucine, the longer side chain of isoleucine increases the steric hindrance with the adjacent β17-sheet, affecting the binding of the upper end of the β17-sheet to FAD (Fig. 6e). In addition, the mutant K351E breaks the hydrogen bond with K299, destroying the locked conformation of the respective loop and α-helix where they were located, and further affects the binding to the substrate or FAD (Fig. 6f).

Our results suggested that although these key residue changes were located on the periphery of the protein and are not directly involved in the recognition and binding of substrates or coenzymes, they could indirectly affect the configuration of the binding cavity through allosteric modification of adjacent residues and, consequently, increase the binding affinity of the substrate or FAD.

### Phylogenetic tree analysis of functional site evolution.

It is well known that the functional sites on proteins are usually highly evolutionarily conserved. To reconstruct the evolutionary trajectory of the Tet(X) active sites, we searched protein sequence databases with the PSI-BLAST program using Tet(X2) as the template sequence and obtained 115 nonredundant Tet(X) sequences (with at least one amino acid difference).

**FIG 7:**
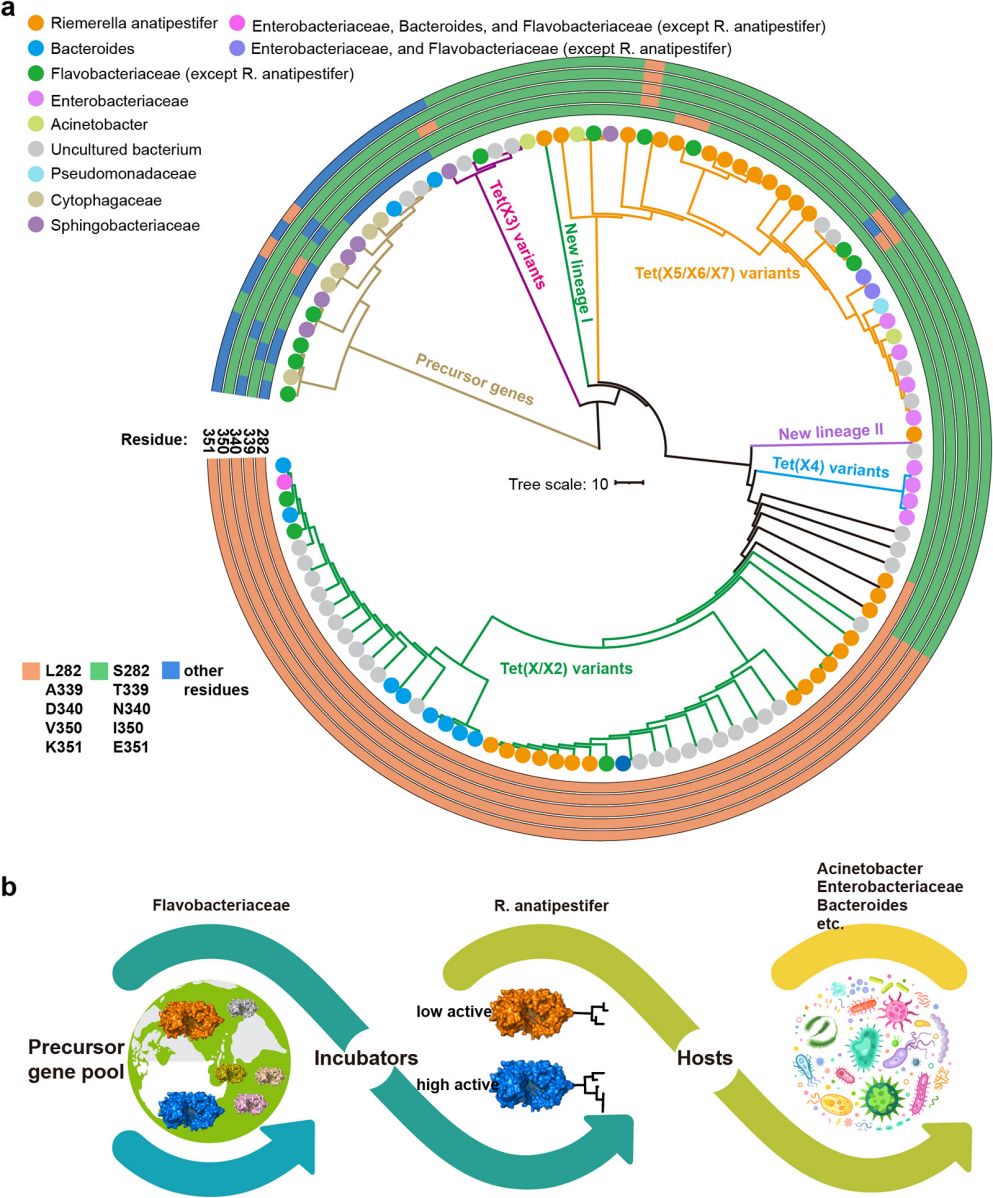
(a) Phylogenetic relationship of 115 unique Tet(X) homologous sequences. The species and the amino acids at the 5 active sites on each sequence are indicated according to the color legend. The 7 major clades are marked with different colors. (b) Schematic diagram of the evolution and spread of *tet*(X) genes.

A phylogenetic tree containing 115 sequences was subsequently constructed. Bayesian analysis of the population structure (BAPS) divided these sequences into 7 major clades, corresponding with Tet(X/X2), Tet(X3), Tet(X4), Tet(X5/X6/X7), precursor gene, and other new lineages (Fig. 7a). The results showed that the Tet(X)/Tet(X2) clade had the largest number of unique sequences (*n* = 39), but no residue changes were observed at any of the 5 active sites. In contrast, most sequences in clades Tet(X3) to Tet(X7) harbor all the five key residue changes, indicating that these five mutants were not unique to Tet(X4).

Recent genomic evolution studies suggested that the family Flavobacteriaceae may be the potential ancestral source of the tigecycline resistance gene *tet*(X) (18, 23). The flavin-dependent monooxygenases carried by *Flavobacteriaceae* bacteria have low amino acid similarity with Tet(X), and some of them have been verified to have no tetracycline degradation function (18). Therefore, these sequences were tentatively designated the precursor genes of Tet(X). Examination of the precursor *Flavobacteriaceae* Tet(X) sequences showed that the distribution of the 5 active residues was relatively diverse, and single or multiple residue changes (S282, T339, N340, I350, and/or E351) that lead to enhanced Tet(X) enzyme activity were found in some sequences, suggesting the likelihood of parallel evolution of Tet(X) in the ancestor and descendant hosts.

Furthermore, of the 115 sequences, 30 were exclusively distributed in R. anatipestifer, accounting for 26% of total sequences, a frequency significantly higher than those in any other species. Further phylogenetic tree analysis showed that these 30 R. anatipestifer sequences were widely dispersed throughout nearly all Tet(X) clades [except for Tet(X3)], and a few were located in some new lineages. Examination of the distribution of the five active residue sites in R. anatipestifer *tet*(X) sequences showed that the allele combinations were highly diverse, including both mixed or complete set of 5 active residue changes (Fig. 7a). These results provided evidence that R. anatipestifer may act as an incubator for Tet(X), leading to high-level tigecycline resistance.

## DISCUSSION

Newer generations of tetracycline antibiotics, including tigecycline, eravacycline, and omadacycline, are regarded as “life-saving” drugs against clinical MDR pathogens, including *bla*_NDM_- and *mcr*-positive strains. Unfortunately, emerging new resistance mechanisms, especially enzymatic antibiotic inactivation, threaten recent progress on bringing these newer generations tetracyclines to the clinic. These highly active Tet(X) orthologs, which confer resistance to tigecycline, eravacycline, omadacycline, and all other classes of tetracycline antibiotics, have now been increasingly detected in clinical and animal isolates; however, knowledge about their active sites leading to high-level resistance and about their evolutionary path remains limited.

In this study, the combination of domain exchange and site-directed mutagenesis experiments identified 5 key residue changes that led to the increase activity of Tet(X2). Importantly, the 5 residue changes were 100% conserved among all tigecycline-resistant Tet(X) orthologs [Tet(X3)-Tet(X7)] (see Fig. S2 in the supplemental material), suggesting that these residue changes played essential roles in molecular evolution of Tet(X) orthologs toward high-level tigecycline resistance. Although previous studies showed that residue changes in the catalytic cavity and the FAD binding cavity were important for the function of Tet(X) (16), the 5 residue changes found in the present study were all located on the outer surface of the protein and were distinct from the catalytic cavity and FAD binding cavity. Structural analysis further suggested that these mutations could potentially interfere with the interaction force with neighboring amino acid residues, resulting in changes of the enzyme spatial configuration and therefore indirectly affecting the dynamics of the substrate and FAD binding cavity. Some previous studies have also showed that the amino acid residues located outside the substrate binding cavity can affect function by modulating protein dynamics (24, 25). Similar examples have been described in other enzymes. For example, amino acid mutations outside the substrate binding cavity of β-lactamases and macrolide kinase can increase the flexibility of the binding pocket, thereby expanding the substrate range or improving catalytic activity (26, 27).

Identification of highly active residue changes in Tet(X) is critical to guide the optimization of novel tetracycline structures and the development of effective Tet(X) enzyme inhibitor combinations that overcome resistance by inactivation. Among clinical pathogens, tigecycline resistance was mostly attributable to ribosomal protection or to high antibiotic efflux expression. The development of newer generations of tetracyclines, e.g., tigecycline, eravacycline, and omadacycline, was partially driven by the idea of overcoming these traditional clinical resistance mechanisms. However, along with the increase in descriptions of plasmid- or chromosome-mediated Tet(X) in various environmental and clinical pathogens, future tetracycline antibiotic development should consider approaches against enzymatic inactivation. A combination of antibiotics and enzyme inhibitor therapy, for example, β-lactam/β-lactamase inhibitor combinations, has become a standard approach for managing resistance by antibiotic inactivation. The clinical application of novel β-lactamase inhibitors (avibactam, vaborbactam, and relebactam) along with β-lactams (ceftazidime, meropenem, and imipenem) demonstrated high efficacy against MDR Gram-negative pathogens. Similarly, recent studies showed that anhydrotetracycline and its semisynthetic analogues were able to inhibit tetracycline destructase enzymes, including Tet(X), by two complementary inhibitory mechanisms, competitive inhibition by blockade of substrate binding and mechanistic inhibition by restraining FAD cofactor dynamics (11, 28, 29). Likewise, novel tetracycline/tetracycline destructase inhibitor combination therapy could serve as a promising strategy to overcome resistance by enzymatic inactivation [e.g., Tet(X)] and restore the use of this important class of antibiotics. Our study also suggested that, besides the substrate and FAD binding sites, the peripheral residues identified in current study can be considered potential candidates for the tetracycline destructase inhibitor design.

Genes encoding tetracycline-degrading enzymes and their homologues are widely present in the natural environment and in human and animal gut metagenomes (30, 31). Tetracycline-degrading enzymes, including Tet(X), are considered to be evolutionarily originated from the environment, while mobile genetic elements promote their transfer and spread into different biological niches (2). Thousands of Tet(X) homologues with >40% amino acid similarity have been found in the NCBI protein database, including numerous sequences from environmental, animal, and human metagenome samples. Although phenotypic resistance was not established for most of these genes, functional selection, including highly active site screening, is critical to properly survey the resistance landscape. The discovery and characterization of these novel environmental resistance genes before they are acquired by clinical pathogens could potentially minimize their clinical impact and inspire proactive approaches to curb emerging resistance.

The identification of functional sites also contributed to better understanding of the evolutionary trajectory of the Tet(X) enzyme family. Interestingly, we found that Tet(X) sequences from R. anatipestifer were widely distributed in different Tet(X) clades, with a high detection rate in comparison with those in other species. In addition, unlike the conservation of the 5 active residues in Tet(X3) to Tet(X7) (see Fig. S2 in the supplemental material), the Tet(X) sequences from R. anatipestifer showed high diversity, with different combinations of residue mutations. R. anatipestifer is an avian pathogen, belonging to the Flavobacteriaceae family, which can cause the characteristic serositis and sepsis of domestic ducks, geese and turkeys (32). R. anatipestifer has a highly plastic genome and can easily obtain foreign genes through natural transformation (32). Recent genomic studies suggested that the *Flavobacteriaceae* family is the origin of the *tet*(X) (23), and our latest research also reasoned that the *tet*(X) genes originated from *Flavobacteriaceae* species and were subsequently spread to environmental and clinical strains such as Acinetobacter spp. and E. coli under the mobilization of IS*CR2* (18). The findings of the current study led us to update our previous hypothesis on the molecular evolution of Tet(X). Our study suggests that R. anatipestifer has played an important role in the molecular evolution of Tet(X). The *tet*(X) ancestor genes from *Flavobacteriaceae* bacteria may have first been transmitted into R. anatipestifer. R. anatipestifer not only acted as a main bridge vector in the transmission of *tet*(X), but also served as a major reservoir for high-resistance Tet(X) mutational changes (Fig. 7b).

In summary, our research revealed the molecular basis of enzyme activity between different Tet(X) orthologs and expanded our understanding of the structural characteristics of Tet(X). These findings are critical to guide the rational design of novel tetracyclines capable of evading enzymatic inactivation, as well as the design of novel inhibitors for use in combination therapies with tetracycline antibiotics. Given the existence of a large number of undefined *tet*(X)-like genes in environmental and human commensal metagenomes, active monitoring of the spread of these genes and understanding of their evolutionary path are critical for proactively managing this emerging resistance mechanism.

## MATERIALS AND METHODS

### Strains and genetic manipulations.

Two E. coli expression systems were used in this study, namely JM109-PBAD24 for determination of MIC and BL21(DE3)-pET28a for protein expression. In addition, the *tet*(X3), *tet*(X4), *tet*(X5), *tet*(X6), and *tet*(X7) genes involved in this study were isolated from strains archived in our laboratory, while the *tet*(X) and *tet*(X2) gene sequences were commercially synthesized. The pBAD24 expression system was constructed as described previously (8). In brief, primers targeting the full length of the Tet(X) gene, along with EcoRI and SalI restriction sites, were designed and used for PCR. The PCR products and the plasmid vector pBAD24 were digested with the restriction endonucleases EcoRI/SalI and then ligated at 16°C overnight following the manufacturer’s instructions (New England Biolabs). Sanger sequencing was used to confirm the correct pBAD24 plasmid constructs. Last, the recombinant plasmid constructs were transferred into E. coli competent JM109 cells by transformation.

For the domain swapping experiment, primers with 20-bp homology arms were used to amplify the N-terminal, middle, and C-terminal domains of Tet(X2) and Tet(X4), and overlap PCRs were used to generate different recombination domain variants. Site-directed mutagenesis was performed according to the QuikChange II site-directed mutagenesis kit manual, using the Tet(X2) sequence as the template. All constructs were verified by Sanger sequencing. All of the primers used in this study are listed in Table S1 in the supplemental material.

10.1128/mSystems.00050-21.1 TABLE S1 Primers used in this study. Restriction sites of EcoRI and SalI enzymes are underlined. Download Table S1, DOCX file, 0.03 MB. Copyright © 2021 Cui et al. 2021 Cui et al. https://creativecommons.org/licenses/by/4.0/ This content is distributed under the terms of the Creative Commons Attribution 4.0 International license.

### Multiple-sequence alignment and structure modeling.

The model structure of Tet(X4) was obtained with Modeller v9.24, using the Tet(X2)-tigecycline complex (PDB identifier 4A6N) as the template; AutoDock v4.2 software was then used to dock small-molecule substrates and the Tet(X2) protein.

### Determination of tetracycline susceptibility.

The MICs of tetracyclines (tetracycline, minocycline, tigecycline, eravacycline, and omadacycline) for constructs were determined using the broth microdilution method. The results were determined in triplicate and repeated on two different days. E. coli ATCC 25922 was used as a quality control strain. Susceptibility results were interpreted according to the guidelines of the European Committee on Antimicrobial Susceptibility Testing (EUCAST) (http://www.eucast.org/clinical_breakpoints).

The method of agar dilution to determine the sensitivity of tigecycline was as follows. In brief, each E. coli construct was transferred into LB broth and cultured at 37°C to an optical density at 600 nm (OD_600_) of 0.6. Then, 10-fold serial dilutions of the bacterial solution were spotted on the LB agar plate containing the desired concentrations of tigecycline. Both the broth dilution method and the agar dilution method were supplied with 0.1% arabinose to maintain the expression of Tet(X) in pBAD24 vector.

### Expression and purification of Tet(X) enzymes.

Different *tet*(X) genes and their domain swapping mutants were ligated into pET28a using the restriction sites EcoRI and BamHI and then transformed into E. coli BL21(DE3) as described above. A single colony was transferred into 5 ml of LB medium supplemented with 50 mg/liter of ampicillin and allowed to shake at 37°C for 12 h. The overnight culture was transferred into 200 ml of fresh LB medium containing kanamycin (50 mg/liter) and incubated at 37°C. Once the OD_600_ of the culture reached 0.4 to 0.6, isopropyl-β-d-thiogalactopyranoside (IPTG) was added at a final concentration of 1 mM and the culture was induced at 16°C for 20 h. Cells were harvested by centrifugation at 6,000 × *g* for 20 min and resuspended in buffer A (20 mM Tris-HCl [pH 7.5] and 500 mM NaCl). The resuspended cells were then lysed by sonication and precipitated by centrifugation of at 10,000 × *g* for 30 min at 4°C, followed by filtering by a 0.45-μm filter membrane. The processed protein mixture was added to Ni-nitrilotriacetic acid (NTA) preequilibrated with buffer A (20 mM Tris-HCl and 500 mM NaCl [pH 8.0]). A linear gradient elution was then applied by the addition of buffer B (20 mM Tris-HCl, 500 mM NaCl, and 500 mM imidazole [pH 8.0]) and the target proteins were collected.

### MALDI-TOF MS analysis.

The matrix-assisted laser desorption ionization–time of flight mass spectrometry (MALDI-TOF MS) analysis was performed as previously reported (33), with minor modifications. In brief, 100 mM N-Tris(hydroxymethyl) methylaminopropane sulfonic acid (TAPS) (pH 7.5), 500 μM NADPH, 50 μM eravacycline, 0.5 mM MgCl_2_, and 1.25 μM enzyme was adjusted to a final volume of 100 μl in an Eppendorf tube. The tube was then mixed and incubated in a metal bath at 37°C for 15 min. After incubation, a 1:3 mixture of hydrochloric acid and acetonitrile was added to stop the reaction. Subsequently, 1 μl supernatant was spotted onto an MSP 384 target polished steel plate (Shimadzu, Kyoto, Japan) and left to dry at room temperature, and then 1 μl matrix (α-cyano-4-hydroxycinnamic acid) was taken to cover the target point. A Shimadzu Performance mass spectrometer and Shimadzu Biotech MALDI-MS software were used to acquire mass spectra in positive linear ion mode operating between 100 and 1,000 Da.

### Spectrophotometric analysis of Tet(X) activity.

Spectrophotometric analysis was performed in a 200 μl Microcon tube containing 100 mM TAPS (pH 7.5), 500 μM NADPH, 50 μM eravacycline, and 0.5 mM MgCl_2_. These compounds were preincubated at 37°C for 5 min prior to the addition of Tet(X) enzymes to initiate the reaction. After the Tet(X) enzymes (1.25 μM) were added, the observance value changes were continuously monitored on a microplate reader (Thermo Fisher Scientific) under the UV-visible (Vis) spectrum (295 to 410 nm) within 10 min at a scanning interval of 60 s. The change of absorbance at 340 nm corresponds with the consumption of NADPH.

### Phylogenetic analysis.

A protein BLAST search with default options was performed using the amino acid sequence of Tet(X2) as a query. With the cutoff values of >70% sequence identity and >90% query coverage, 115 unique Tet(X)-like sequences were obtained. ClustalW was used to perform multiple-sequence alignment. Phylogeny reconstruction was inferred using the maximum-likelihood method (MEGA v7.0). The best-fitting model selected by IQ-Tree was JTT. The initial tree for the heuristic search was obtained automatically by applying the maximum-parsimony method. A discrete gamma distribution was used to model evolutionary rate differences among sites (5 categories [+G, parameter = 1.4016]). The analysis involved 115 amino acid sequences. All positions with less than 95% site coverage were eliminated. There was a total of 375 positions in the final data set. The tree was visualized in iTOL v.4 (34).

### Data availability.

Source data underlying the main text and Fig. 5c can be found in Data Set S1. The pdb format file of the Tet(X4) homology model can be found at https://doi.org/10.6084/m9.figshare.14529693.v1.

10.1128/mSystems.00050-21.4 DATA SET S1 Source data of the UV full-wavelength scanning experiment (see Fig. 5c). Download Data Set S1, XLSX file, 0.1 MB. Copyright © 2021 Cui et al. 2021 Cui et al. https://creativecommons.org/licenses/by/4.0/ This content is distributed under the terms of the Creative Commons Attribution 4.0 International license.
