# Supplementary figures and images for: Evolutionary Trajectory of the Tet(X) Family: Critical Residue Changes towards High-Level Tigecycline Resistance
Source: mSystems. 2021 May 18;6(3):e00050-21. doi: 10.1128/mSystems.00050-21 (PMC8269203; doi:10.1128/mSystems.00050-21)

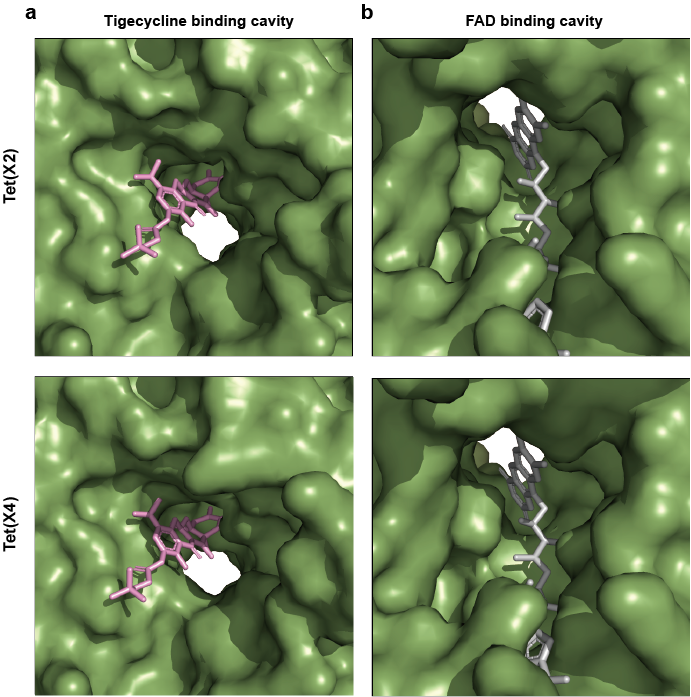

Supplement: FIG S1 [file msystems.00050-21-sf001.tif]

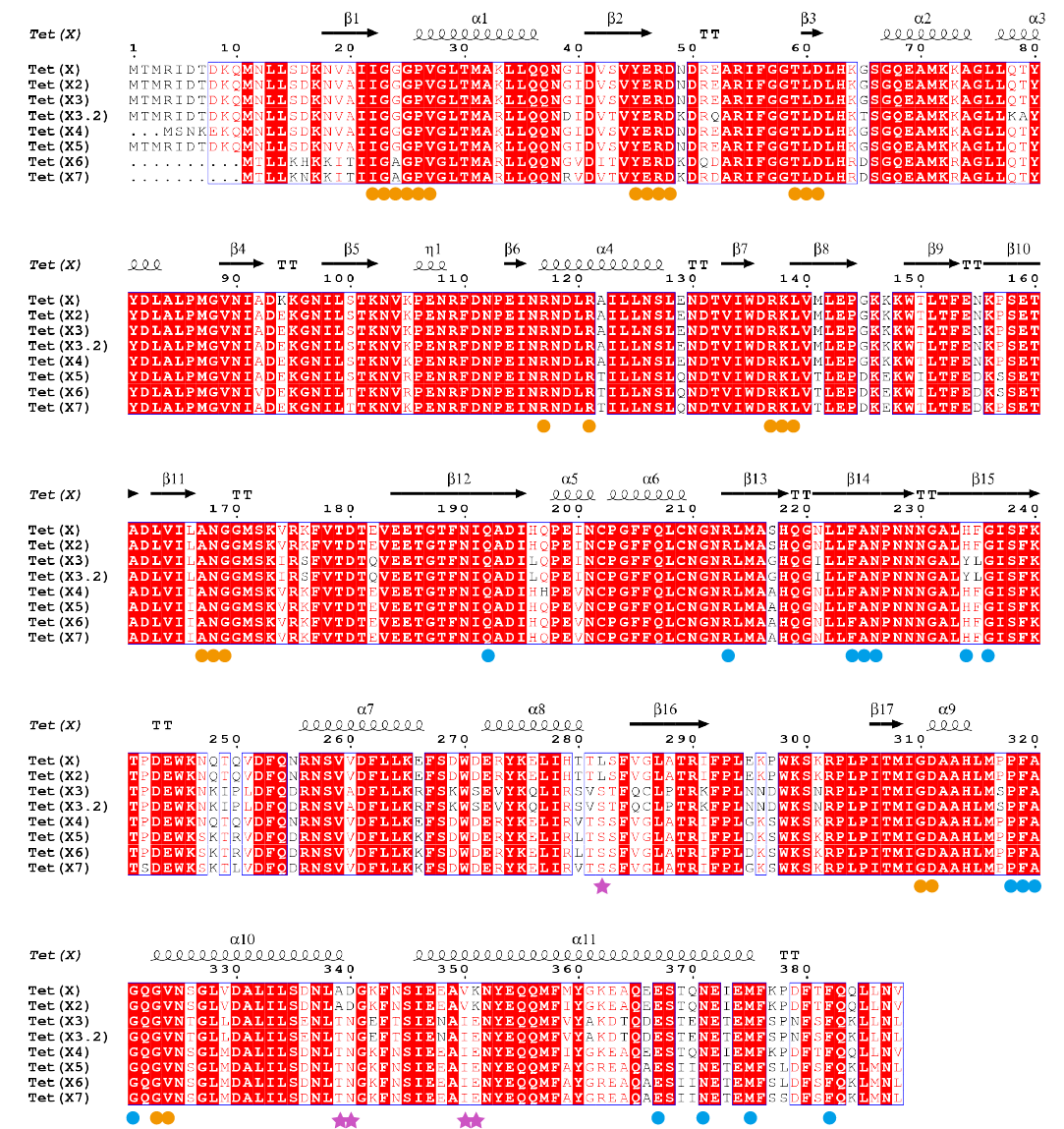

Supplement: FIG S2 [file msystems.00050-21-sf002.tif]
