# Supplementary material for: Evolutionary Trajectory of the Tet(X) Family: Critical Residue Changes towards High-Level Tigecycline Resistance
Source: mSystems. 2021 May 18;6(3):e00050-21. doi: 10.1128/mSystems.00050-21 (PMC8269203; doi:10.1128/mSystems.00050-21)
Supplement: TABLE S1 [file msystems.00050-21-st001.docx]

| Primer | Nucleotide sequence (5’ to 3’) | Size (bp) | Reference |
| --- | --- | --- | --- |
| Tet(X2)L282S-F | CGACGTCATCATTTGTAGGATTG | 5683 | This study |
| Tet(X2)L282S-R | CCTACAAATGATGACGTCGTATGAATCA |  |  |
| Tet(X2)A339T-F | GTCTGATAATCTAACCGATGGAAAAT | 5683 | This study |
| Tet(X2)A339T-R | CATCGGTTAGATTATCAGACAATATC |  |  |
| Tet(X2)D340N-F | CTGATAATCTAGCCAATGGAAAATTTAA | 5683 | This study |
| Tet(X2)D340N-R | TCCATTGGCTAGATTATCAGACAATATCA |  |  |
| Tet(X2)V350I-F | GAAGAGGCTATTAAAAATTATGAACAG | 5683 | This study |
| Tet(X2)V350I-R | TAATTTTTAATAGCCTCTTCAATGCTAT |  |  |
| Tet(X2)K351E-F | GAAGAGGCTGTTGAAAATTATGA | 5683 | This study |
| Tet(X2)K351E-R | TAATTTTCAACAGCCTCTTCAATGCTATT |  |  |
| Tet(X4)S279L-F | GATTCGTGTGACATTGTCTTTTGTAGGGTT | 5674 | This study |
| Tet(X4)S279L-R | AAGACAATGTCACACGAATCAGTTCTTTGTAG |  |  |
| Tet(X4)T336A-N337D-F | TTGTCGGATAATCTGGCCGATGGGAAATTTAACAG | 5674 | This study |
| Tet(X4)T336A-N337D-R | AAGACAATGTCACACGAATCAGTTCTTTGTAG |  |  |
| Tet(X4)I347V-E348K-F | CAGCATTGAAGAGGCTGTTAAAAATTATGAACAGC | 5674 | This study |
| Tet(X4)I347V-E348K-R | AGCCTCTTCAATGCTGTTAAATT |  |  |
| n-terminal-F | TACGCGAATTCATGACAATGCGAATAGATACAGAC |  | This study |
| n/N-terminal-R | GTCGTTTTCTAAACTATTCAACAAGAT |  |  |
| N-terminal-F | TACGCGAATTCATGAGCAATAAAGAAAAACAAATGAATTTAC |  | This study |
| m/M-terminal-F | ATCTTGTTGAATAGTTTAGAAAACGAC |  |  |
| m-terminal-R | CCTACAAATGACAACGTCGTATG |  | This study |
| M-terminal-R | CCTACAAAAGATGATGTCACACGAATC |  |  |
| t-terminal-F | ATTCGTGTGACATCATCTTTTGTAGGATTGGCTACACGGAT |  | This study |
| t/T-terminal-R | TACGCGTCGACTTATACATTTAACAATTGCTGAAACGT |  |  |
| T-terminal-F | ATTCATACGACGTTGTCACTTTTGTAGGGTTAGCGACACGA |  | This study |
